# Associations between Life's Essential 8 scores and systemic immune-inflammation index among American-adult populations without cardiovascular disease

**DOI:** 10.3389/fmed.2025.1588835

**Published:** 2025-08-05

**Authors:** Shan-Shan Huang, Xiao-Shuang Yu, Tong Lin, Zhen-Ye Xie, Hai-Yan Mao, Zhou-Xin Yang

**Affiliations:** ^1^Department of Critical Care Medicine, Ningbo Medical Center Lihuili Hospital, Ningbo, China; ^2^Department of Otolaryngology, Jiangshan People's Hospital, Quzhou, China; ^3^Zhejiang Key Laboratory of Geriatrics and Geriatrics Institute of Zhejiang Province, Zhejiang Hospital, Hangzhou, China

**Keywords:** Life's Essential 8, systemic immune-inflammation index, cardiovascular health, American-adult populations, cross-sectional study

## Abstract

**Background:**

Cardiovascular health (CVH) profoundly impacts human health and quality of life. Increasing evidence suggests a close association between cardiovascular disease (CVD) and systemic immune-inflammatory levels. This study explores the potential correlation between Life's Essential 8 (LE8) scores and the systemic immune-inflammation index (SII), a novel immune-inflammatory index among US adults. This study provides evidence supporting the role of systemic inflammation reduction in promoting CVH.

**Methods:**

Utilizing data from the National Health and Nutrition Examination Survey (NHANES) spanning 2007–2018, we investigated information from 21,403 adult participants. Participants were categorized into low CVH (0–49), moderate CVH (50–79), and high CVH (80–100) groups based on LE8 scores. We employed weighted linear regression analysis and subgroup analysis, along with restricted cubic spline curves (RCS) to explore the association between LE8 and SII and the dose-response relationship.

**Result:**

A significant negative correlation was found between higher LE8 scores and lower SII levels. Compared to the low CVH group, the β coefficients for SII in the CVH moderate and CVH high groups were −40.02 (95% CI: −58.99 to −21.05, *p* < 0.001) and −77.62 (95% CI: −102.4 to −52.80, *p* < 0.001), respectively. Additionally, both LE8 scores and health behaviors scores showed a significant linear negative correlation with SII. There was an inverted “U-shaped” non-linear relationship between health factors scores and SII, and the health factor score was 284.724, with a maximum SII threshold of 518.010 (1,000 cells/μl). The health factor score is positively associated with SII below 518.010 and negatively associated above this threshold. Subgroup analyses showed that the negative association was stable in most subgroups. The negative correlation was insignificant among those aged >65 and Mexican Americans.

**Conclusion:**

LE8 showed a significant negative correlation with SII. The findings suggest that maintaining higher LE8 scores to some extent promotes CVH and helps alleviate systemic inflammation, potentially benefiting overall health.

## 1 Introduction

To improve cardiovascular health (CVH) in populations, the American Heart Association (AHA) introduced a new concept of CVH in 2010, along with measures for assessing and monitoring it, known as Life's Simple 7 (LS7) (1). LS7 is based on seven health behaviors and factors, including diet, physical activity (PA), smoking, blood glucose, blood lipids, blood pressure (BP), and body mass index (BMI) (2). With increasing research, poor sleep health was identified as being associated with an increased risk of cardiovascular mortality (3–6). Studies suggest that sleep affects BP, inflammation, glucose homeostasis, and other factors related to CVH (7–9), highlighting the potential importance of sleep for overall health and cardiometabolic health. Therefore, in 2022, AHA introduced a new CVH score, Life's Essential 8 (LE8), consisting of four main health behaviors and health factors. The four health behaviors include diet, PA, smoking state, and sleep and the four health factors include BMI, BP, blood lipids, and blood glucose (10). Both LS7 and LE8 aim to promote individual and population CVH. It has been suggested that when CVH is optimized, the aforementioned behaviors and factors are associated with longer cardiovascular disease (CVD)-free survival, overall life expectancy, and higher quality of life (11–13).

The development of CVD is closely related to inflammation and the immune system. Neutrophils are effectors of the innate immune response and regulate processes such as autoimmunity and chronic inflammation (14). Platelets maintain homeostasis, are involved in mediating acute and chronic inflammatory processes, and contribute to the inflammatory environment (15). Lymphocytes are the key cells of the adaptive immune response that link the innate and adaptive immune responses (16). A novel immune-inflammatory index, the systemic immune-inflammation index (SII), was first proposed by Hu et al. (17) and defined as “platelet ^*^ neutrophil count/lymphocyte count.” It reflects the balance between inflammation and immune response, with elevations suggesting an increased inflammatory state of the disease and a weakened immune response. Previous studies have shown that elevated SII is strongly associated with CVD. For instance, research has demonstrated a significant association between higher SII and increased risk of CVD, as well as its prognosis and mortality rates (18–20). SII has been identified as a risk factor for atrial fibrillation (21), systolic insufficiency in cardiomyopathies (22), and may serve as an independent predictor for massive pulmonary embolism (23). In addition, studies have found that SII is an independent risk factor for the development of coronary heart disease (CHD) in patients with non-alcoholic fatty liver disease (NAFLD) and is closely associated with the prediction and severity of CHD (24). Furthermore, SII has a certain predictive value for the increased risk of all-cause and cardiovascular mortality in adults with hypertension (25).

This study utilized a nationally representative cohort with diverse racial backgrounds, employing the complex multi-stage probability sampling design of the National Health and Nutrition Examination Survey (NHANES) database. Explore the relationship between LE8 scores and SII. Elaborate on whether maintaining optimal CVH status can improve systemic inflammation and potentially reduce the occurrence of various other diseases.

## 2 Methods

### 2.1 Data source and study participants

All data for this study were obtained from the NHANES in the US. Information regarding the study design and data from the NHANES database can be publicly accessed at https://www.cdc.gov/nchs/nhanes/. Briefly, the NHANES database comprises five main categories of data, including demographic data, dietary data, examination data, laboratory data, questionnaire data, and restricted access data. It employs a complex, multi-stage, probability sampling method, providing extensive information on the nutrition and health of the general U.S. population, with all survey participants consenting to participation. The NHANES study has obtained approval from the National Center for Health Statistics (NCHS) Research Ethics Review Board, and detailed information regarding the approval of the NCHS Research Ethics Review Board can be accessed on the NHANES website (https://wwwn.cdc.gov/nchs/nhanes/default.aspx).

**Figure 1:**
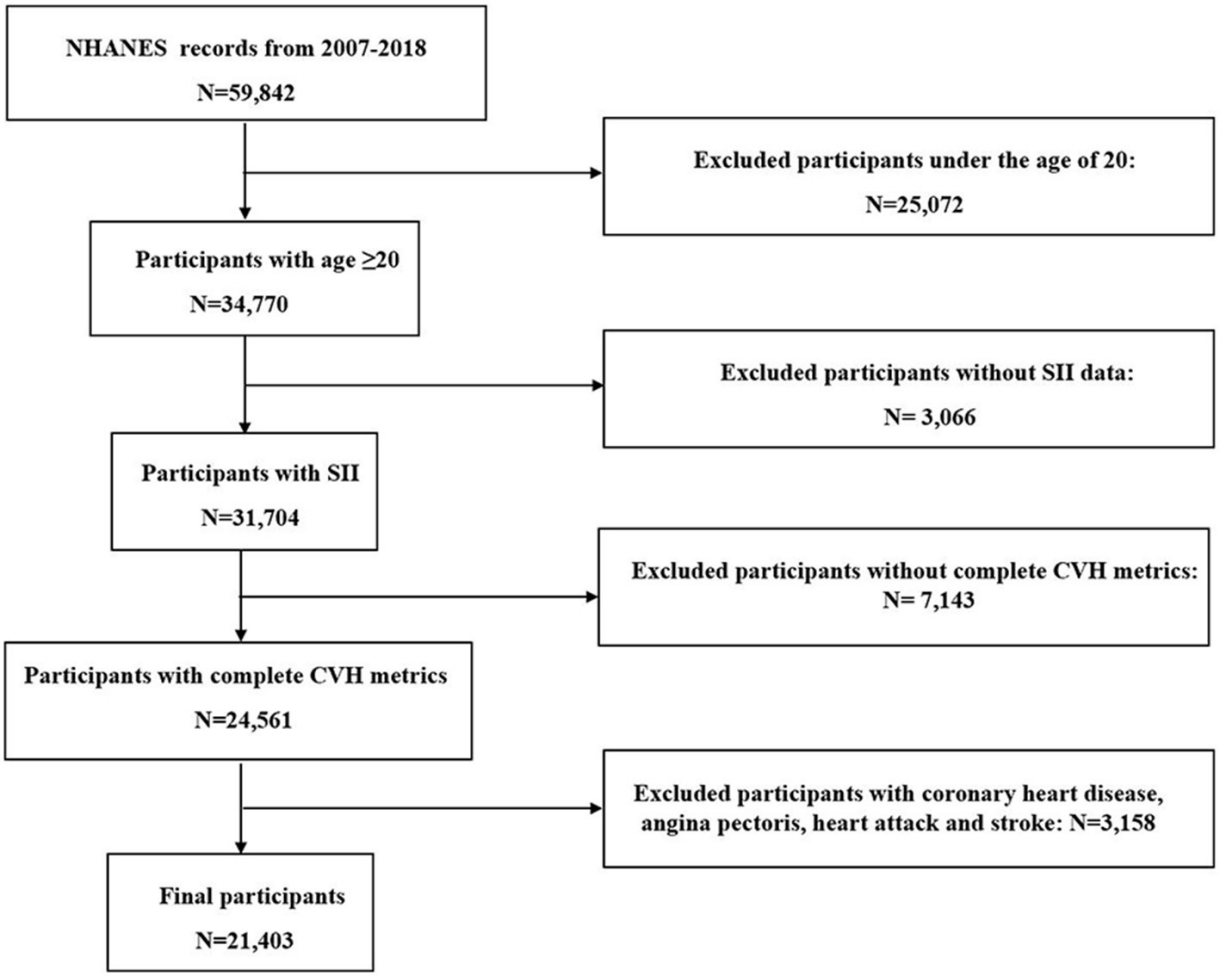
Flowchart of participants. SII, systemic immune-inflammation index; CVH, cardiovascular health.

This study included data from NHANES from 2007 to 2018, totaling six consecutive cycles with a total of 59,842 participants. Adult participants with complete data on LE8 scores, SII, etc. were mainly included in this study. Figure 1 shows the screening flowchart of this study. After applying the exclusion criteria, the following participants were excluded from this study: (1) 25,072 participants younger than 20 years of age; (2) 3,066 participants with missing SII data; (3) 7,143 participants who lacked complete CVH data; and (4) 3,158 individuals with self-reported coronary heart disease, angina pectoris, heart attack, and stroke were excluded. A final total of 21,403 participants were included.

### 2.2 Measurement of LE8 and SII

LE8 comprises eight indicators, divided into health behaviors (diet, PA, smoking state, and sleep) and four health factors (BMI, BP, blood lipids, and blood glucose). These eight items are scored according to publicly available official calculation methods. Each item is scored on a scale of 0–100 points, and the total score is derived from the unweighted average of the eight indicators. Based on the final LE8 score, CVH is categorized into three groups: low (0–49), moderate (50–79), and high (80–100) (11). Among them, the dietary indicator is evaluated based on the Healthy Eating Index-2015 (HEI-2015) and includes only participants with 2-day dietary data for this study (26, 27). Additionally, BMI and BP are obtained from physical examination data. PA, smoking state, and sleep are collected through self-reported questionnaires. Blood lipids and blood glucose were obtained from laboratory data. Detailed algorithms for calculating LE8 scores for various indicators in NHANES data can be found in previous literature (28).

The platelet count, neutrophil count, and lymphocyte count used to calculate the SII are obtained from laboratory data of whole blood cell counts. Whole blood cell counts are measured by the NHANES Mobile Examination Center (MEC) using the Beckman Coulter DxH 800 instrument and are expressed in units of ( × 1,000 cells/μl). The specific formula for calculating the SII follows previous literature and is defined as platelet count × neutrophil count/lymphocyte count (17).

### 2.3 Covariates

Based on previous research experience (29, 30), potential relevant covariates included in the analysis are age, poverty impact ratio (PIR), gender (male, female), race/ethnicity (Mexican American, Non-Hispanic Black, Non-Hispanic White, Other Hispanic, and Other/multiracial), white blood cell count (WBC), neutrophil count, platelet count, lymphocyte count, blood potassium, blood sodium, creatinine, albumin, alanine aminotransferase (ALT), aspartate aminotransferase (AST), urea nitrogen, total calcium, lactate dehydrogenase (LDH), phosphorus, total bilirubin, and uric acid.

### 2.4 Statistical method

According to the NHANES database analysis guidelines, weighted methods were employed to reduce data variability for statistical analysis (weight parameter: WTDR2D/6). The normality of the independent and dependent variables was first verified using the adoption of the Lilliefors test; the results showed that both the independent and dependent variables were non-normally distributed. Continuous variables were represented using weighted means and standard deviations, and group comparisons were made using the Kruskal–Wallis *H* test. Categorical variables were presented as unweighted counts and weighted percentages, with group comparisons conducted using the chi-square test. Three models were utilized: Model 1 (unadjusted), Model 2 (adjusted for age, gender, PIR, and race), and Model 3 (adjusted for age, gender, PIR, race, creatinine, albumin, ALT, AST, urea nitrogen, total calcium, LDH, phosphorus, total bilirubin, and uric acid). The dependent variable was SII, and weighted linear regression analyses were performed to assess the strength of the variables' associations with LE8, its components, and CVH three groups (low, moderate, and high), calculating β coefficients. Potential non-linear relationships between SII and LE 8 scores, health behaviors, and health factors were explored by comparing model fit metrics at different nodes after using RCS (three nodes) and using likelihood ratio tests. Subsequently, subgroup analyses were performed based on age, gender, race, and PIR, visually represented using forest plots. All data analyses were conducted using SPSS 26.0 and R 4.2.3, with statistical significance set at *p* < 0.05.

## 3 Results

### 3.1 Baseline characteristics of the study population

Table 1 shows the baseline characteristics of this study population, comprising a total of 21,403 participants. Based on LE8 scores, individuals were categorized into low CVH (0–49), moderate CVH (50–79), and high CVH (80–100) groups. It was observed that the high CVH group consisted predominantly of females and mostly non-Hispanic White individuals. Additionally, they exhibited younger age and higher income levels. As expected, individuals with higher CVH scores showed lower levels of SII, along with lower neutrophil count, platelet count, white blood cell count, lymphocyte count, creatinine, AST, ALT, and LDH. Conversely, total bilirubin and blood phosphorus levels were higher in this group.

**Table 1:** Weighted characteristics of the participants by CVH groups.

**Characteristic**	**Overall, *n* = 21,403^a^**	**CVH low, *n* = 4,085^a^**	**CVH moderate, *n* = 14,249^a^**	**CVH high, *n* = 3,069^a^**	***p*-value^b^**
Age (year)	46.11 (16.39)	51.39 (14.28)	46.74 (16.68)	39.41 (14.80)	< 0.001
**Gender**					
Female	11,284 (52.14%)	2,082 (48.77%)	7,344 (50.62%)	1,858 (60.32%)	< 0.001
Male	10,119 (47.86%)	2,003 (51.23%)	6,905 (49.38%)	1,211 (39.68%)	
PIR	3.06 (1.65)	2.49 (1.62)	3.06 (1.64)	3.56 (1.57)	< 0.001
**Race**					
Mexican American	3,272 (8.96%)	635 (9.05%)	2,268 (9.51%)	369 (6.95%)	< 0.001
Non-Hispanic Black	4,432 (10.59%)	1,154 (15.99%)	2,924 (10.81%)	354 (5.24%)	
Non-Hispanic White	9,085 (66.85%)	1,641 (63.50%)	5,974 (66.03%)	1,470 (72.57%)	
Other Hispanic	2,255 (5.85%)	397 (5.36%)	1,588 (6.26%)	270 (4.82%)	
Other/multiracial	2,359 (7.75%)	258 (6.09%)	1,495 (7.38%)	606 (10.42%)	
LE8 scores	65.37 (14.93)	42.01 (6.32)	65.05 (8.27)	86.30 (4.52)	< 0.001
PA scores	51.59 (46.98)	9.53 (26.45)	49.89 (46.62)	93.21 (19.02)	< 0.001
Smoke scores	67.97 (40.95)	38.45 (42.94)	67.97 (40.50)	92.99 (17.31)	< 0.001
Sleep scores	83.59 (23.83)	68.88 (30.24)	84.42 (22.58)	93.16 (14.19)	< 0.001
BP scores	70.30 (32.00)	45.35 (32.29)	70.04 (30.76)	92.37 (16.34)	< 0.001
Diet scores	39.37 (14.76)	32.92 (12.43)	38.59 (14.08)	47.62 (15.33)	< 0.001
BMI scores	60.37 (33.74)	33.69 (29.51)	59.20 (32.23)	87.10 (19.82)	< 0.001
Non-LDL scores	64.52 (30.89)	43.34 (29.85)	63.70 (29.41)	85.36 (22.50)	< 0.001
Glucose scores	84.71 (24.50)	63.56 (29.44)	86.12 (22.84)	97.65 (9.84)	< 0.001
SII (μmol/L)	531.95 (326.49)	586.96 (357.35)	533.93 (335.61)	478.37 (249.18)	< 0.001
WBC (1,000 cells/μl)	7.30 (2.83)	8.36 (5.10)	7.25 (2.14)	6.56 (1.81)	< 0.001
Neutrophils (1,000 cells/μl)	4.32 (1.72)	5.01 (1.97)	4.29 (1.67)	3.84 (1.46)	< 0.001
Platelet count (1,000 cells/μl)	245.10 (62.14)	257.48 (72.34)	245.37 (61.49)	233.62 (52.17)	< 0.001
Lymphocyte (1,000 cells/μl)	2.17 (1.80)	2.44 (4.17)	2.15 (0.80)	2.00 (0.60)	< 0.001
Creatinine (ml/min)	76.96 (27.31)	78.99 (34.18)	77.16 (27.30)	74.55 (19.48)	< 0.001
Albumin (g/dl)	42.84 (3.43)	41.77 (3.50)	42.83 (3.39)	43.78 (3.22)	< 0.001
ALT (U/L)	25.18 (17.33)	29.09 (20.78)	25.41 (17.50)	21.11 (11.78)	< 0.001
AST (U/L)	25.10 (13.66)	26.72 (15.74)	24.98 (13.83)	24.11 (10.76)	0.006
Urea nitrogen (mmol/L)	13.45 (4.99)	13.68 (5.82)	13.45 (4.93)	13.27 (4.39)	0.708
Total calcium (mg/dl)	9.40 (0.36)	9.41 (0.38)	9.40 (0.36)	9.40 (0.33)	0.624
LDH (U/L)	131.72 (29.09)	137.74 (32.22)	132.27 (29.04)	124.71 (24.76)	< 0.001
Phosphorus (mg/dl)	3.75 (0.57)	3.73 (0.61)	3.74 (0.56)	3.80 (0.55)	< 0.001
Total bilirubin (mg/dl)	0.65 (0.32)	0.59 (0.27)	0.65 (0.32)	0.72 (0.33)	< 0.001
Uric acid (mg/dl)	5.40 (1.39)	5.90 (1.45)	5.44 (1.37)	4.86 (1.22)	< 0.001

^a^Mean (SD); n (unweighted; %).

^b^Wilcoxon rank-sum test for complex survey samples; chi-squared test with Rao & Scott's second-order correction.

CVH, cardiovascular health; PIR, poverty impact ratio; LE8, Life's Essential 8; PA, physical activity; BP, blood pressure; BMI, body mass index; LDL, low-density lipoprotein cholesterol; SII, Systemic Immune-Inflammation Index; WBC, white blood cell; ALT, alanine aminotransferase; AST, aspartate aminotransferase; LDH, lactate dehydrogenase.

### 3.2 Association between LE8 Score and SII

We established three models: Model 1 (unadjusted), Model 2 (adjusted for age, gender, PIR, and race), and Model 3 (adjusted for age, gender, PIR, race, creatinine, albumin, ALT, AST, urea nitrogen, total calcium, LDH, phosphorus, total bilirubin, and uric acid), and conducted weighted linear regression analysis and RCS. As shown in Table 2, the LE8 score was analyzed as a continuous variable. The results revealed a significant negative correlation between SII levels and LE8 scores [β95% CI: −2.379 (−2.843 to −1.916), *p* < 0.001]. The β coefficient for LE8 scores was −2.379 (1,000 cells/μl/point), indicating that an increase of one unit in SII was associated with a decrease of 2.379 points in LE8 scores. This negative correlation remained significant after controlling for relevant covariates [Model 2 and Model 3; β95% CI: −2.657 (−3.087 to −2.227), *p* < 0.001; β95% CI: −2.029 (−2.539 to −1.520), *p* < 0.001].

**Table 2:** Weighted linear regression analysis of SII and LE8 scores, LE8 component scores, and LE8 scores groups.

**Characteristic**	**Model 1**		**Model 2**		**Model 3**	
	β **(95% CI)**	***p*** **-value**	β **(95% CI)**	***p*** **-value**	β **(95% CI)**	***p*** **-value**
LE8 scores	−2.379 (−2.843, −1.916)	< 0.001	−2.657 (−3.087, −2.227)	< 0.001	−2.029 (−2.539, −1.520)	< 0.001
PA scores	−0.578 (−0.710, −0.446)	< 0.001	−0.513 (−0.640, −0.385)	< 0.001	−0.410 (−0.541, −0.279)	< 0.001
Smoke scores	−0.243 (−0.391, −0.096)	0.001	−0.356 (−0.511, −0.201)	< 0.001	−0.333 (−0.493, −0.173)	< 0.001
Sleep scores	−0.192 (−0.518, 0.135)	0.247	−0.294 (−0.561, −0.026)	0.032	−0.173 (−0.451, 0.105)	0.218
BP scores	−1.179 (−1.566, −0.792)	< 0.001	−1.428 (−1.811, −1.045)	< 0.001	−0.938 (−1.342, −0.535)	< 0.001
Diet scores	−0.577 (−0.760, −0.393)	< 0.001	−0.555 (−0.765, −0.344)	< 0.001	−0.414 (−0.651, −0.177)	< 0.001
BMI scores	−0.760 (−0.951, −0.568)	< 0.001	−0.842 (−1.047, −0.638)	< 0.001	−0.409 (−0.688, −0.130)	0.005
Non-LDL scores	−0.286 (−0.473, −0.098)	0.003	−0.212 (−0.405, −0.020)	0.031	−0.126 (−0.334, 0.081)	0.230
Glucose scores	−0.757 (−1.065, −0.448)	< 0.001	−0.770 (−1.049, −0.492)	< 0.001	−0.509 (−0.789, −0.230)	< 0.001
**CVH**						
Low	Reference		Reference		Reference	
Moderate	−53.02 (−72.51, −33.54)	< 0.001	−52.57 (−71.35, −34.15)	< 0.001	−40.02 (−58.99, −21.05)	< 0.001
High	−108.60 (−132.20, −84.97)	< 0.001	−107.20 (−129.80, −84.56)	< 0.001	−77.62 (−102.40, −52.80)	< 0.001

Model 1: Unadjusted; Model 2: Adjusted for age, gender, PIR, and race; Model 3: Adjusted for age, gender, PIR, race, creatinine, albumin, ALT, AST, urea nitrogen, total calcium, LDH, phosphorus, total bilirubin, and uric acid.

CI, confidence interval; CVH, cardiovascular health; PIR, poverty impact ratio; LE8, Life's Essential 8; PA, physical activity; BP, blood pressure; BMI, body mass index; LDL, low-density lipoprotein cholesterol; ALT, alanine aminotransferase; AST, aspartate aminotransferase; LDH, lactate dehydrogenase.

Further analysis of the individual components of LE8 scores revealed that SII was significantly negatively correlated with PA, smoke, BP, diet, BMI, and glucose scores (*p* < 0.001). Among these, BP scores contributed the most, while there was no significant correlation with sleep or non-high-density-lipoprotein cholesterol (non-LDL) scores (*p* > 0.05). Additionally, when stratified by LE8 scores and compared with the low CVH group, the β coefficients of SII in the moderate CVH group were −53.02 (95% CI: −72.51 to −33.54, *p* < 0.001), and in the high CVH group were −108.60 (95% CI: −132.2 to −84.97, *p* < 0.001). This indicates that SII levels were significantly lower in the moderate CVH group and high CVH group than in the low CVH group, and were lowest in the high CVH group. This correlation remained significant after controlling for relevant covariates (*p* < 0.001). These findings suggest that higher LE8 scores are associated with lower SII levels, demonstrating a negative correlation.

**Figure 2:**
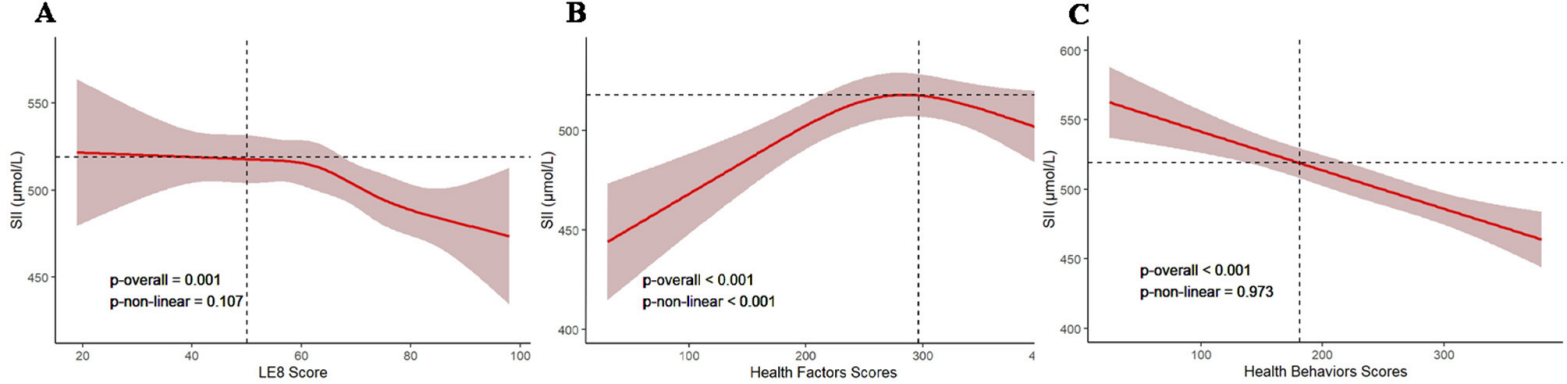
Dose-response relationship curves between SII and adjusted β values for LE8 scores, health factors scores, and health behaviors scores. **(A)** LE8 scores, **(B)** health factors scores, **(C)** health behaviors scores. The model was adjusted for age, gender, PIR, race, creatinine, albumin, ALT, AST, urea nitrogen, total calcium, LDH, phosphorus, total bilirubin, and uric acid. SII, systemic immune-inflammation index; LE8, Life's Essential 8; PIR, poverty impact ratio; ALT, alanine aminotransferase; AST, aspartate aminotransferase; LDH, lactate dehydrogenase.

To explore the potential non-linear relationship and dose-response relationship between SII and LE8 scores, we conducted RCS analysis in Model 3, examining the association of SII with LE8 scores, health factor scores, and health behavior scores. As shown in Figure 2, the *p*-values for non-linearity between SII and LE8 scores (Figure 2A) and health behavior scores (Figure 2C) were both >0.05, indicating no non-linear relationship between SII and LE8 scores or health behaviors scores. However, interestingly, there was a non-linear relationship between SII and health factors scores (Figure 2B, *p* < 0.05), displaying a reverse “U” shape, and the health factor score was 284.724, with a maximum SII threshold of 518.010 (1,000 cells/μl). The health factor score is positively associated with SII below 518.010 and negatively associated above this threshold.

### 3.3 Subgroup analysis

**Figure 3:**
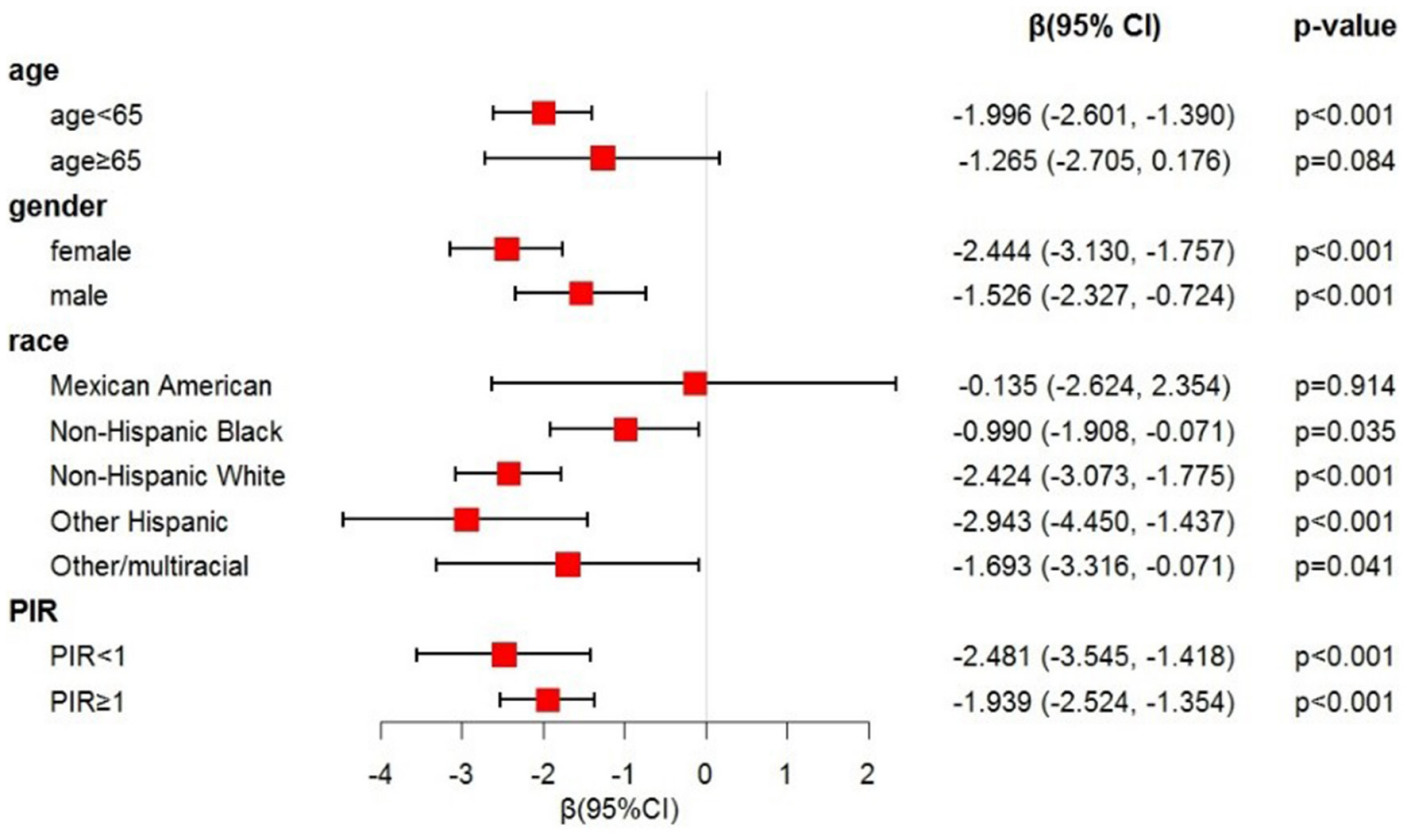
Forest plot for subgroup analysis. The model was adjusted for age, gender, PIR, race, creatinine, albumin, ALT, AST, urea nitrogen, total calcium, LDH, phosphorus, total bilirubin, and uric acid. PIR, poverty impact ratio; ALT, alanine aminotransferase; AST, aspartate aminotransferase; LDH, lactate dehydrogenase.

To assess the stability of this negative association, we performed subgroup analyses for age, gender, race, and PIR. In Figure 3, stratification by gender and PIR revealed significant correlations between SII and LE8 scores in all subgroups. After stratifying by age, significant correlations were observed only among participants aged < 65 years, but not in those aged >65 years. When stratified by race significant associations were observed in participants of all races except Mexican American.

## 4 Discussion

In this study, we found a significant negative correlation between SII levels and LE8 scores. Even after controlling for relevant covariates, this negative correlation remained significant, indicating that higher SII levels were associated with lower LE8 scores. The LE8 scores encompass four health behaviors scores and four health factors scores. Interestingly, there was no non-linear relationship between SII levels and LE8 scores or health behavior scores. In contrast, a reverse “U-shaped” non-linear relationship existed with the four health factors scores, further suggesting that when SII > 518.010 (1,000 cells/μl), higher SII levels were associated with lower scores in health factors, potentially leading to a decline in CVH status. Linear regression analysis indicated that among these four health factors scores, BP made the greatest contribution. We further conducted subgroup analyses by age, gender, race, and PIR, and the results showed that this negative correlation remained stable in most strata. However, it was not significant in individuals aged >65 years old or those of Mexican American ethnicity, suggesting a cautious interpretation of these results in these populations. In conclusion, this study suggests that SII may be a readily accessible and valuable indicator for assessing CVH status in non-CVD populations.

CVD is a leading cause of global mortality and disability. According to reports from 2019, CVD accounted for over 18 million deaths worldwide, approximately one-third of all global deaths. High systolic BP, dietary risks, high and low-density lipoprotein cholesterol, air pollution, high body mass index, smoking, high blood sugar, and renal dysfunction are major risk factors for CVD (31). A higher CVH state is associated with lower CVD risk, with mechanisms involving inflammation, endothelial function, atherosclerosis, cardiac stress and remodeling, hemostatic factors, and epigenetics (13, 32, 33). Studies suggest that low-grade chronic inflammation increases the risk of atherosclerosis and insulin resistance, leading to persistent low-level immune system activity (34). Furthermore, patients with chronically immune-mediated inflammatory diseases have an increased risk of CVD (35). The immune system and inflammatory processes play a crucial role in the pathogenesis of atherosclerosis (36).

SII, as a novel indicator quantifying systemic immune and inflammatory responses, incorporates indices of platelets, neutrophils, and lymphocytes. Previous research has primarily focused on the relationship between SII and CVD. For instance, elevated SII has been associated with increased CVD risk and mortality rates (18–20, 37, 38). Additionally, higher SII levels increase the risk of hemorrhagic and ischemic stroke subtypes as well as overall mortality (39). However, there has been limited research on the association between the LE8 scores and SII. Therefore, this study, after excluding relevant cardiovascular-related diseases, explored for the first time the relationship between LE8 scores and its components with SII among adults. The findings indicated a significant negative correlation between SII and both LE8 scores and its components, suggesting that maintaining optimal CVH status may improve systemic inflammation.

LE8, as a comprehensive indicator, considers not only health factors such as BP and lipids but also health behaviors like sleep, nicotine exposure, and exercise. Previous studies have shown that sleep-related disorders in adults, such as sleep duration, sleep problems, high risk of obstructive sleep apnea (OSA), and daytime sleepiness, are associated with higher levels of SII (40). Our study found a significant negative linear correlation between SII and health behavior scores, and although there was a negative association between SII and sleep scores, this association was not statistically significant. This may be related to differences in the methods used to assess sleep scores. Additionally, smoking status is believed to influence the relationship between SII and metabolic syndrome (41). In our study, smoking score showed a significant negative correlation with SII, suggesting that smoking not only affects CVH status but may also impact systemic inflammation. Therefore, our study suggests that in adults without CVD, interventions such as smoking cessation, improving diet, regular exercise, and controlling blood glucose and BP may be more effective in reducing inflammation in individuals with low LE8 scores. It's noteworthy that a non-linear relationship exists between SII and health factors scores, which may suggest a deeper connection between SII and overall CVH. Although this study couldn't pinpoint the exact relationship between SII and the four health factors, when SII = 518.010 (1,000 cells/μl), it may suggest that maintaining these four health factors at optimal levels could be more beneficial for maintaining CVH status.

Subgroup analysis in this study indicated that the association between SII and LE8 scores remained stable in most subgroups. However, in the subgroup analysis stratified by age, the relationship between SII and LE8 scores was not significant among individuals aged over 65 years. This suggests that, after adjusting for confounding factors, the elevated SII levels in individuals aged over 65 are not significantly associated with CVH status. Firstly, the declining immune system in the older adult leads to increased senescent cells, causing systemic inflammation (42). Additionally, with aging, there is a mild pro-inflammatory state termed inflammaging. On one hand, aging characteristics exhibit mild pro-inflammatory states referred to as aging-associated inflammatory responses (43, 44). On the other hand, senescent cells contribute to the production of pro-inflammatory cytokines, collectively known as the senescence-associated secretory phenotype (45). These mechanisms may weaken the correlation between SII and LE8 scores. In the subgroup analysis based on race, this relationship was not significant among Mexican Americans, which may be partly related to their environmental and genetic risk factors predisposing them to a high-risk status for CVD (46, 47).

Our study has several strengths. First, we are the first to evaluate the relationship between SII and LE8, utilizing dose-response analyses to more effectively illustrate the associations between SII, LE8, health behaviors, and health factors. Second, compared to other commonly used inflammatory markers, such as C-reactive protein (CRP) or interleukin-6, SII serves as a low-cost biomarker with promising clinical utility. Third, our study leveraged NHANES data, which employs a complex, multistage probability sampling design and accounts for relevant confounding factors, ensuring a more representative analysis. This enhances the applicability of our findings to broader populations.

Nevertheless, certain limitations should be acknowledged. To begin with, this study is a cross-sectional design based on a U.S. population, and the use of longitudinal analyses or causal modeling would be more conducive to revealing the deeper mechanisms underlying the study's finding of an association between LE8 and SII. This is particularly critical as systemic inflammation can both influence and be influenced by cardiovascular health, emphasizing the need for further investigation into the underlying mechanisms. Second, the measurements of key variables such as lifestyle habits in the study were only self-reported, which has questionable reliability and validity, and the quality of the study could be further improved by using more objective measures. Moreover, SII was measured or calculated at a single time point, whereas dynamic monitoring over time might provide more accurate insights in clinical settings. Finally, while this study adjusted for multiple covariates, unmeasured confounding factors, such as socioeconomic or genetic variables, may still have influenced the results.

## 5 Conclusion

This study demonstrates a negative linear correlation between SII and health behavior, health factors, and LE8 scores. Additionally, there exists a non-linear relationship between SII and health factors, displaying a reverse “U” shape. These findings suggest that maintaining optimal LE8 scores not only promotes CVH status but also helps alleviate systemic inflammation, thereby potentially benefiting overall health. However, the causal mechanisms underlying these associations require further investigation and elucidation.

## Data Availability

The original contributions presented in the study are included in the article/supplementary material, further inquiries can be directed to the corresponding authors.
